# Impact of routine birth early infant diagnosis on neonatal HIV treatment cascade in eThekwini district, South Africa

**DOI:** 10.4102/sajhivmed.v21i1.1084

**Published:** 2020-06-02

**Authors:** Vidya Kalawan, Kevindra Naidoo, Moherndran Archary

**Affiliations:** 1 Department of Paediatrics and Children Health, University of KwaZulu-Natal, Durban, South Africa; 2 King Dinizulu Hospital, Durban, South Africa; 3 Maternal Adolescent and Child Health (MatCH), University of the Witwatersrand, Johannesburg, South Africa; 4 King Edward VIII Hospital, Durban, South Africa

**Keywords:** early infant diagnosis, birth HIV testing, HIV PCR, treatment cascade, paediatrics, LMIC

## Abstract

**Background:**

Early infant diagnosis (EID) of human immunodeficiency virus (HIV) and early initiation of antiretroviral therapy (ART) in HIV-infected infants can reduce the risk of mortality and improve clinical outcomes. Infant testing guidelines in KwaZulu-Natal, South Africa, changed from targeted birth EID (T-EID) only in high-risk infants to a routine birth EID (R-EID) testing strategy in 2015.

**Objectives:**

To describe the impact of the implementation of R-EID on the infant treatment cascade.

**Method:**

A retrospective analysis of a facility-based clinical database for the eThekwini district and the National Health Laboratory Services (NHLS) was conducted. All data on neonates (< 4 weeks of age) diagnosed with HIV between January 2013 and December 2017 (T-EID [2013–2015] and R-EID [2016–2017]) were extracted including follow-up until 1 year post-diagnosis.

**Results:**

A total of 503 neonates were diagnosed HIV-infected, with 468 (93.0%) initiated on ART within a median of 6 days. There was a significant increase in the estimated percentage of HIV-infected neonates diagnosed (21% vs. 86%, *p* < 0.001) and initiated on ART (90% vs. 94.3%, *p* < 0.001) between the T-EID and R-EID periods. Despite achieving over 90% of HIV-infected neonates diagnosed and initiated on ART in 2017, retention in care and viral suppression remained low.

**Conclusion:**

Implementation of R-EID in eThekwini district improved diagnosis and initiation of ART in HIV-infected neonates and should be recommended as part of diagnostic guidelines. These gains are, however, lost because of poor retention in care and viral suppression rates and therefore required urgent attention.

## Introduction

Early infant diagnosis (EID) of human immunodeficiency virus (HIV) and initiation of antiretroviral therapy (ART) are essential in reducing morbidity and mortality in HIV-infected infants.^1,2,3,4,5,6,7,8,9,10^ In 2018, the Joint United Nations Programme on HIV/AIDS (UNAIDS) reported that 89% of HIV-exposed infants were tested before 8 weeks of age. However, only 63% of HIV-infected children under 14 years were on ART in South Africa.^11^ Strategies to increase the uptake of EID and linkage to ART care are essential in achieving the UNAIDS 95-95-95 targets for testing, treatment and viral suppression.

In 2013, the World Health Organization (WHO) recommended an HIV nucleic acid polymerase chain reaction (PCR) assay at 4–6 weeks of age, or at the earliest opportunity for EID in resource-limited settings, although birth HIV PCR testing was a conditional recommendation.^12^ Despite the improved capacity for EID, only half of all HIV-exposed infants were tested within 2 months of life in the 21 African Global Plan countries.^13^ Multiple factors contribute to the low rates of early testing of HIV-exposed infants, including the lack of testing and/or sample collection sites, stock-outs of HIV testing commodities at facility and central laboratory levels, poorly functioning sample transport networks, and delays and gaps in returning results to carers.^14,15,16,17,18^ Access to timely HIV diagnosis for HIV-exposed infants is a critical step to close the treatment coverage gap and reduce HIV-associated mortality for children.^19^

South Africa’s national HIV management guideline was revised in June 2015, with the change in the EID recommendation from targeted birth EID (T-EID) testing in high-risk infants to a routine birth EID (R-EID) testing for all HIV-exposed neonates, in addition to virological testing at 10 or 18 weeks.^20^ High-risk infants include all premature (born before 37 weeks’ gestational age), low-birth-weight (LBW < 2500 g) or symptomatic HIV-exposed neonates, or those born to women who were un-booked or received a late diagnosis of HIV or received < 4 weeks of ART, or had viral loads (VLs) of > 1000 copies/mL.^21^ Universal birth testing of all HIV-exposed infants is simpler to implement than targeted birth testing.^21^ Within 1 year of implementation of birth testing, the national birth testing coverage exceeded 90%.^22^

eThekwini district, KwaZulu-Natal (KZN), has used an electronic data management system (Tier.Net) to record all patient data at a facility level to monitor HIV and ART services and provides a system of monitoring and evaluating the ART programme. A retrospective analysis of routinely collected data from the Tier.Net database over the period of January 2013–December 2017 was carried out. The aim was to determine the proportion of infants with a positive HIV PCR who are successfully initiated on ART and to evaluate the time taken to ART initiation before and after the initiation of birth EID in eThekwini district, South Africa. This study also evaluated the immunological and virological response and outcomes at 12 months after ART initiation.

## Methods

The databases of the facility-based patient management system (Tier.Net) and the National Health Laboratory Services (NHLS) were retrospectively analysed to identify all infants diagnosed HIV positive at birth or less than 4 weeks of age between January 2013 and December 2017 at any facility in eThekwini district. Date of ART initiation and clinical and laboratory investigations for the first year after ART initiation were further extracted.

The Tier.Net system is a facility-based patient management system implemented in 2011 by the South African National Department of Health to monitor patients accessing ART from public health facilities. Data on all infants (< 1 year of age) diagnosed and/or initiating ART during the study period were extracted onto a password-protected Excel spreadsheet from Tier.Net.

The Tier.Net data were first filtered to include neonates and then compared with the NHLS EID district report by the investigator to ensure accuracy and to remove duplications. The database included demographic information (e.g. age, sex and facility), age at ART initiation, CD4 T-lymphocyte count and HIV VL results from 1 year after ART initiation. In addition, all biochemical laboratory test results were collected. The database was de-identified prior to analysis. The NHLS performed all laboratory tests as part of the standard of care by using standard commercial laboratory kits.

eThekwini district is the largest district in KZN with a population of 3.7 million and includes urban, peri-urban and semi-rural populations. An estimated 1.9 million people are living with HIV in KZN, with 34% living in eThekwini district.^23^ Categorical variables were described by percentages, and continuous variables were described by medians and interquartile ranges (IQRs). Univariate analysis of characteristics associated with HIV diagnosis, ART initiation and viral suppression was performed by using Fisher’s exact test or the Mantel–Haenszel test for categorical variables. The analysis was stratified into T-EID (from 2013 to 2015) and R-EID (from 2016 to 2017) periods. *p* values < 0.05 were considered significant. All two-way interactions were evaluated by using STATA IC version 15.

The estimate of the number of HIV-infected neonates in eThekwini was calculated by using the total number of live births in the respective years from Statistics South Africa with 41% of these neonates being HIV-exposed based on the HIV antenatal seroprevalence survey, and an in-utero HIV transmission rate of 0.9%.^24,25^

### Ethical consideration

A patient consent waiver was obtained for this study because of the retrospective nature of the database audit. Ethical approval for the study was obtained from the University of KwaZulu-Natal Biomedical Research Ethics Committee (BREC Ref No: BE023/18), and it was approved by the KwaZulu-Natal Department of Health (HRKM Ref: 183/18, NHRD Ref: KZ_201805_010) and eThekwini district (Ref: 22/02/18).

## Results

During the study period, 4049 HIV-infected infants were captured from the Tier.Net database in eThekwini district, and 503 were less than 4 weeks of age at diagnosis. There was a female predominance within the R-EID infants (318/503, 63.2%). A total of 468 (93%) neonates were initiated on ART in the first month of life. Of the 224 (44.5%) infants who initiated ART within the first month of life and those remained in care at 1 year of age, 91 (40.6%) infants had a documented HIV VL. Fifty-six (61.5%) infants had a VL of <1000 copies/mL and 25 (27.5%) infants had a VL of < 50 copies/mL.

Differences between the T-EID and R-EID periods are shown in Table 1. The number of neonates initiating ART increased from 135 (90%) in the T-EID period to 333 (94.3%) in the R-EID (*p* = 0.081). Median age at ART initiation was at 6 days of life (IQR 0–16) overall. In the R-EID period, the HIV VL was significantly lower and fewer patients were lost to follow-up (Table 1).

**TABLE 1:** Characteristics of the cohort of 503 neonates with confirmed human immunodeficiency virus infection.

Variable	Pre-birth EID (2013–2015) (*n* = 150)		Post-birth EID (2016–2017) (*n* = 353)		*p*
	*n*	%	*n*	%	
Gender (female)	101	67.3	217	61.5	0.450
Number of initiations	135	90	333	94.3	0.081
Age at start of ART (days)†	5.00	0.00–16.8	7.00	0.00–15.3	0.598
Baseline CD4 T-lymphocyte counts†	228	141–375	229	0.685–558	0.002
Pre-treatment viral load†	558 000	969–1040 000	124	46.0–3440	0.046
Died (number or %)	7	4.7	9	2.5	0.226
Lost to follow-up (number or %)	72	48	124	35.1	0.0067
Transferred (number or %)	19	12.7	48	13.6	0.779

ART, antiretroviral therapy; EID, early infant diagnosis.

† , Median (interquartile range).

Viral suppression in patients with a VL available at 1 year remained low in both periods, with 6 (0.8%) versus 19 (4.6%) infants who were retained in care having a VL of < 1000 copies/mL in the T-EID and R-EID periods, respectively (*p* < 0.005). Similarly, 7 (1.0%) versus 34 (8.0%) infants who were retained in care had a VL of < 50 copies/mL in the T-EID and R-EID periods, respectively (*p* < 0.005).

By using the Division of AIDS (DAIDS) Table for Grading the Severity of Adult and Paediatric Adverse Events, we found 12 infants with a low haemoglobin level, eight with grade 3 and two with grade 4. Four infants had grade 1 alanine aminotransferase levels during the study period.

The DAIDS toxicity table is internationally recognised. It contains parameters or adverse events with severity grading guidance.^26^

**FIGURE 1:**
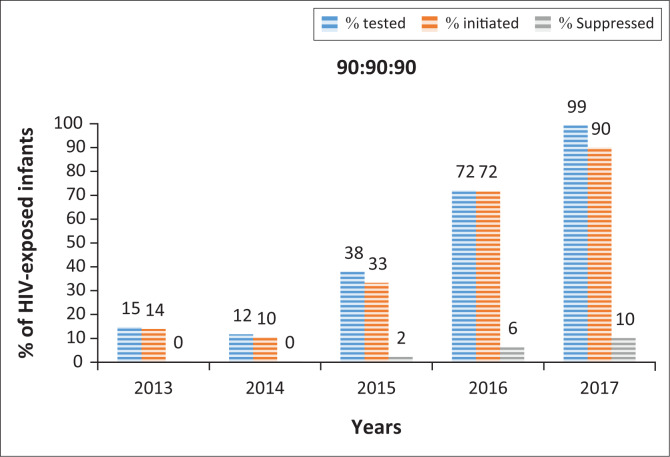
Graph representing the numbers of patients tested for human immunodeficiency virus, initiated on treatment and suppressed at 1 year of age from 2013 to 2017 in eThekwini district.

On the basis of the estimated number of HIV-infected neonates born in eThekwini district during the study period, there was a significant increase in the percentage of neonates tested (20.7% vs. 85.7%, *p* < 0.001) and initiated on ART (90.0% vs. 94.3%, *p* < 0.001) between the T-EID and R-EID periods (Figure 1). In 2017, the percentage of neonates tested and initiated on ART had reached above 90%; however, the percentage of infants with viral suppression (HIV VL < 1000 copies/mL) at 1 year remained low at 10%.

## Discussion

In this retrospective analysis of all HIV-infected neonates diagnosed within the first 4 weeks of age and initiated on ART in eThekwini district over 5 years, we found a significant increase in the percentage of neonates diagnosed and initiating ART with the introduction of routine birth EID; however, viral suppression at 1 year after ART initiation remained low.

Achieving the WHO 90:90:90 targets for the infant treatment cascade is vital for improving the outcomes of HIV-infected infants. Because of a lack of evidence, the WHO conditionally recommends the addition of birth testing to existing EID strategies to identify HIV infection in HIV-exposed infants. In a study modelling the impact of the implementation of birth EID, a positive impact on both uptake and timing of ART initiation in HIV-infected infants was predicted.^27^ Several studies have reported both increases in the number of HIV-exposed infants tested and earlier age of HIV diagnosis in programmes that have implemented birth HIV testing.^28,29^

In this study, we found a significant increase in the percentage of infants infected during the peripartum and intrauterine period following the implementation of R-EID compared with during the T-EID in KZN. The prevention of mother-to-child transmission (PMTCT) guidelines in South Africa changed in 2013 with the implementation of Option B+ resulting in more HIV-infected women maintained on ART and retained in the PMTCT cascade.^30^ However, Option B+ was consistently implemented throughout the study period and would not account for the differences noted. Targeted birth EID for high-risk HIV-exposed infants is challenging to implement programmatically, because of the complexity in stratifying according to risk profile in often busy and under-resourced facilities.

Earlier diagnosis of HIV, through the R-EID approach, facilitates improved linkage to care and higher rates of ART initiation. In KZN, the majority of deliveries occur in healthcare facilities with labour wards where birth PCR is performed. In contrast, infant follow-up occurs in well-baby clinics, often at different facilities. Linkage to care is vitally important, and the process starts with obtaining patient contact details in an HIV PCR register when performing the birth PCR following delivery, providing a linkage form to facilitate the transfer of information between the labour ward and well-baby clinics, and active identification facilitated by weekly electronic lists of HIV PCR results supplied by the NHLS directly to key healthcare workers at the facility, district and province. Although a central laboratory for HIV PCR testing is the current testing model in KZN, this may be difficult in areas with logistic problems in tracking, transporting samples and returning the results to the facility and patients. The availability of point-of-care (POC) HIV PCR testing is feasible in several healthcare settings in sub-Saharan Africa and would further improve linkage to care.^31,32^

Early ART initiation in HIV-infected infants decreases the HIV reservoir and improves morbidity and mortality in these children. In this study, the lower baseline HIV VL and higher CD4 count at ART start during the R-EID period is a finding similar to other studies and likely reflects the very early identification of asymptomatic HIV-infected infants. A study conducted in Zambia between 2006 and 2016 also noted better HIV care and a decrease in the average time from diagnosis to treatment initiation from 220 days in 2006 to 9 days in 2015.^33^ Increased exposure to antiretroviral drugs through maternal ART and infant prophylaxis is an additional factor that may have contributed to the lower baseline HIV VL. The performance of POC HIV PCR testing in patients with low-level HIV viraemia is an area of concern resulting in diagnostic dilemmas and delays in ART initiation.^34^

Despite a significant reduction in the number of patients lost to follow-up during the R-EID period, the percentage of patients virally suppressed remained low at 1 year. This finding is similar to other cohorts describing ART initiation during the first 4 weeks of life despite the lower baseline HIV VLs.^35,36^ Bad tasting liquid lopinavir–ritonavir may contribute to the poor VL suppression.

The challenges associated with improving viral suppression and retention in care require urgent attention to fully realise the benefits of earlier HIV diagnosis and ART initiation seen with R-EID. These strategies include improving patient tracking and linkage to care by utilising community health workers and mobile Health applications, for example a messaging system to remind carers of follow-up appointments.^31^ HIV stigmatisation and discrimination can interrupt adherence to treatment and retention in care. Therefore, support is required from local organisations that are involved with HIV education, advocacy groups, faith-based organisations and members of support groups for people living with HIV.^37,38^ Strengthening of referral networks, possibly through the use of an improved patient-held record that is used at all ART facilities, may assist mothers and infants to continue care elsewhere and assist providers at different sites to coordinate care.^39^

An interesting finding was the disproportionate number of HIV-infected women than men identified. Previous studies show that female sex is more susceptible to intrauterine HIV infection.^40^ Women are infected by viruses of lower replication capacity in utero than men, but have a higher interferon (IFN) expression and accelerated antiviral immune response to the virus, which is IFN-resistant.^40^ The possible mechanism underlying this increased female susceptibility is that the sex difference in in-utero infection is most marked in the setting of recent maternal infection. Recently infected mothers are more likely to harbour IFN-resistant virus and women in utero, because of their higher level of immune activation, are more susceptible to the virus.^40^

## Limitations

There are several limitations of the study and the findings because of the retrospective analysis of the data. The facility-based Tier.Net data were dependent on the completeness of the data entered by the facility and may have potentially missed infants not registered. Many patients were captured in the Tier.Net database system that could not be located on the NHLS system despite by using at least two identifiers. Further, as data were limited to eThekwini district, patients who self-transferred or those transferred to other provinces or districts were lost to follow-up. This likely contributed to the observed high loss to follow-up rates. Many patients who were still in care did not have an HIV VL performed at 1 year, further limiting the conclusions related to viral suppression rates in this patient population.

## Conclusion

HIV-infected infants can be identified at birth and ART can be initiated within the first 7 days of life. Despite most infants in our cohort starting ART early, the retention of patients in care was suboptimal and viral suppression was very low. Major challenges moving forward are improving retention in care and viral suppression. Adequate maternal counselling during antenatal care is essential and needs to include a clear explanation about EID and infant ART. This discussion needs to continue through each step in the care cascade.
